# LoRaCELL-Driven IoT Smart Lighting Systems: Sustainability in Urban Infrastructure

**DOI:** 10.3390/s24020574

**Published:** 2024-01-16

**Authors:** Iago Z. Biundini, Milena F. Pinto, Leonardo M. Honório, Miriam A. M. Capretz, Amanda O. Timotheo, Mario A. R. Dantas, Priscilla C. Villela

**Affiliations:** 1 Department of Electrical Engineering, Federal University of Juiz de Fora, Juiz de Fora 36036-900, Brazil; iago.biundini@engenharia.ufjf.br (I.Z.B.); mario.dantas@ice.ufjf.br (M.A.R.D.); 2 Department of Electronics Engineering, Federal Center for Technological Education of Rio de Janeiro, Rio de Janeiro 20271-110, Brazil; milena.pinto@cefet-rj.br; 3 Department of Electrical and Computer Engineering, Western University, London, ON N6A 5B7, Canada; mcapretz@uwo.ca; 4 CEB Lajeado, Brasilia 71215-902, Brazil; priscilla.villela@ceb.com.br

**Keywords:** IoT-enabled smart lighting, LoRaCELL, urban development and sustainability

## Abstract

In recent years, the rate of urbanization has increased enormously, precipitating an escalating demand for improved services and applications in urban areas to improve the quality of life. In the Internet of Things (IoT)era, cities are transforming into smart urban centers. These cities incorporate connected devices, such as intelligent public lighting systems, to enhance their urban infrastructure. Therefore, this work explores the transformative potential of an IoT-enabled smart lighting system in urban environments, emphasizing its essential role in enhancing safety, economy, and sustainability. In this sense, LoRaCELL (Long-Range Cell) is introduced. LoRaCELL is an innovative system that utilizes edge devices for data collection, such as light intensity, humidity, temperature, air quality, solar ultraviolet radiation, ammeter, and voltmeter. It stands as a pioneering solution for intelligent public lighting systems, contributing to advancing IoT-driven urban development. The outcomes showed that the proposed system could successfully synchronize the devices with each other and send IoT sensing data at a low cost compared to traditional technologies such as LoRaWAN.

## 1. Introduction

With the advent of the Internet of Things (IoT), cities worldwide are transforming into smart cities, where urban infrastructure is enhanced with connected devices to improve the quality of life for citizens and the efficiency of urban services. This evolution involves enhancing urban infrastructure with interconnected devices to elevate the quality of life for citizens and the efficiency of urban services. Crucial elements to lay the groundwork for a smart city include various sensors, supporting technologies, and background environments. These components are actively implemented in urban areas, serving as the foundational framework that aligns with the broader vision of creating intelligent and responsive urban environments. One sector that has benefited from this revolution is public lighting. It is possible to remotely monitor and control street lighting through sensors and communication technologies, optimizing energy consumption and enhancing public safety [1,2].

Public lighting plays a crucial role in urban infrastructure, influencing not only the aesthetics of a city but also its safety and economy. As urban areas grow, the demand for efficient and effective public lighting solutions becomes increasingly paramount [3,4]. One of the primary purposes of public lighting is to ensure the safety of citizens. Well-lit streets deter criminal activities and reduce the risk of accidents, especially during nighttime. Pedestrians and drivers rely on adequate lighting to navigate the streets safely [5]. Inadequate or inconsistent lighting can lead to hazardous zones within the city, increasing the potential for accidents and endangering citizens.

With the global shift towards sustainability, cities are pressured to adopt environmentally friendly solutions. Traditional lighting systems consume significant energy and have a shorter lifespan. Transitioning to energy-efficient alternatives, such as light-emitting diode (LED) lighting, reduces a city’s carbon footprint and decreases maintenance costs due to the longer lifespan of these lighting solutions [6]. Given the multifaceted impact of public lighting on cities, several studies have been conducted in this domain by exploring innovative solutions, understanding the needs of different urban areas, and integrating intelligent technologies that can revolutionize how cities approach public lighting [7,8,9].

As the number of things or devices to be connected grows, their connectivity becomes an important issue [10]. In recent years, several studies have explored the use of IoT to enhance public lighting in smart cities. For instance, Leccisi et al. [11] showed a platform to collect, handle, organize, and evaluate the dynamic and static strategic data of urban energy-intensive infrastructure (public lighting, public buildings, e.g., schools), to provide a constant and dynamic assessment of their functional and energetic performances. Rossi et al. [12] proposed a low-cost IoT public lighting control system to exploit cellular networks and cloud computing architectures. Moreover, IoT-based intelligent lamppost systems have been developed to provide smart infrastructure, communicating environmental parameters and images to IoT servers [2,3]. In [2], the authors developed an architecture based on edge and fog computing to realize smart infrastructure, such as lighting systems, environmental parameters, and image sensing in real time. The authors of [3] showed the development of a smart device connected to the lighting system for power quality monitoring and energy-efficient illumination control.

### 1.1. Contributions

As can be seen, much effort has been proposed to address the growing challenges associated with the increasing number of connected devices, especially in enhancing public lighting in smart cities through the IoT [10]. This work proposes the Long Range Cell (LoRaCELL) to contribute to IoT-based urban infrastructure systems by introducing a unified hardware approach for edge devices and gateways, ensuring compatibility with existing LoRaWAN servers. This developed system supports multiple gateways per region, enhancing scalability and efficient data transmission in urban settings. Utilizing LoRa N2P communication, the system integrates with AWS, MongoDB v7.0, and Apache Spark v3.5.0 for robust data handling and advanced analysis. The inclusion of Power BI enables user-friendly data visualization, facilitating informed decision-making and urban planning based on concrete, data-driven insights. LoRaCELL’s holistic approach addresses hardware simplification, scalability, efficient communication, advanced analytics, and user-friendly data visualization. In essence, the main features of the proposed LoRaCELL can be summarized as follows:

- Development of hardware applicable to all devices, edge devices, or gateways. This approach is designed to integrate with existing LoRaWAN servers. This simplifies the system architecture and reduces hardware complexity.
- Support for multiple gateways per region, aligning with the cellular communication concept. This scalable solution ensures efficient data transmission and collection, even in densely populated urban areas, enhancing the overall system performance.
- Implementation of Power BI for data visualization and interpretation. This visualization aids informed decision-making and urban planning based on concrete data-driven insights.

### 1.2. Organization

This article is structured to provide readers with a comprehensive understanding of the research, methodologies, and findings related to the LoRaCELL system and its applications in IoT and smart cities. The rest of this paper is summarized as follows. Section 2 briefly introduces related studies to offer foundations for prior research and developments in the field. Section 3 serves as the foundation of the article, detailing the methodological approach and the materials used in the research. Furthermore, it offers insights into the research design, data collection, and analysis techniques. The end of this section describes the connection and data flow from AWS to MongoDB and subsequently to Power BI, emphasizing the research’s data management and visualization aspects. Section 4 presents the research findings, showcasing the data, analyses, and outcomes derived from the methodologies employed. Finally, Section 5 provides the concluding remarks and future improvements to the proposed LoRaCELL.

## 2. Related Works

Integrating diverse data sources like current, voltage, lighting, UV index, and sound can pose significant challenges. Different researchers have provided innovative solutions to address these challenges [13]. For instance, Zhu et al. [14] explored a hybrid sensing paradigm that combines IoT and crowd sensing. In this approach, users are incentivized to provide data not covered by IoT sensors and to offer feedback for calibrating IoT sensing. This method ensures user privacy by physically obscuring IoT sensors and employing a privacy-preserving protocol for user–server interactions. Meanwhile, Anand et al. [15] evaluated the feasibility of federated learning (FL) in the context of a smart city street light monitoring application. FL significantly improves communication costs and privacy preservation, making it a viable solution for IoT applications in smart cities. In the work of Joy et al. [16], the authors proposed a system for mobile privacy-preserving crowdsourced data collection in smart cities. The system ensures end-to-end security for data exchanges, requiring a management and control side channel. Mante [17] discussed an IoT data processing system for smart cities based on the oneM2M standards [18]. The system employs a multi-tenant architecture with multiple logical databases for efficient data management and uses open APIs to avoid data silos and enable secure data sharing.

Other researchers have also contributed to advancing the field of data integration and LoRaWAN-based IoT applications. Povalac et al. [19] conducted an in-depth analysis of LoRaWAN traffic across four European cities. Their study highlighted significant issues in current LoRaWAN deployments, including security lapses and potential breaches of spectrum regulations. Kane et al. [20] presented a real-world performance comparison between Wi-Fi HaLow and LoRa, specifically in the context of the smart grid. Their findings suggest that Wi-Fi HaLow offers promising performance when juxtaposed with LoRa. Safi et al. [21] introduced a fault-tolerant fire detection system using LoRaWAN in smart buildings, showcasing its reliability in detecting various hazards.

Farhad and Pyun [22] provided a comprehensive survey on the application of machine learning (ML) techniques to enhance the performance of the LoRaWAN network, particularly in resource management. An interesting review of machine learning approaches can be found in [23]. Giuliano et al. [24] explored adaptive transmission algorithms tailored for batteryless IoT sensors that utilize the LoRa protocol. Their work offers insights into optimizing the operation of such devices, considering renewable energy sources. Kumar et al. [25] proposed a networked intelligent gas sensor system (N-IGSS) that leverages the long-range (LoRa) protocol for real-time airborne pollution hazard detection and monitoring. Andreadis et al. [26] discussed deploying IoT devices in remote areas lacking terrestrial Internet connectivity. They introduced a synchronization protocol for opportunistic communication between LoRa IoT devices and an unmanned aerial vehicle (UAV)-based gateway.

Table 1 summarizes the differences between the proposed approach, LoRaCELL, and the prior studies in the field of IoT and data integration, offering a comprehensive perspective on the evolution and innovations in the field of IoT-enabled smart lighting and data integration.

The AURORA system, proposed by Rossi et al. [12], depends on cellular networks, which may be inadequate in areas with weak coverage. LoRaCELL overcomes this by employing LoRa technology, independent of extensive cellular infrastructure, offering a more cost-effective and less complex solution. The integration of IoT-sensing and crowdsensing shown by Zhu et al. [14] and Anand et al. [15] could encounter issues in data precision and user involvement. Note that the device proposed in this work simplifies this process, ensuring efficient data transmission without the complexities associated with these approaches.

The system proposed by Joy et al. [16], while focusing on privacy, might introduce complexities and high costs. Povalac et al. [19] discussed the scalability challenges of LoRaWAN traffic. The high price of LoRaWAN gateways and potential signal blockages in dense urban areas increase costs. Our LoRaCELL approach, with its lower-cost gateways, offers optimized allocation and better performance in smaller regions.

Kane et al. [20] studied Wi-Fi HaLow versus LoRa in smart grids and highlighted cost-effectiveness concerns for smart city lighting. Farhad et al. [22] worked on enhancing LoRaWAN with machine learning, presenting complexity challenges. LoRaCELL offers a viable economic solution for urban lighting systems and simplifies this using LoRa directly, thus avoiding the complexities and costs associated with advanced machine learning techniques.

In LoRaCELL, the primary limitation is the connectivity capacity per gateway. Each gateway, designed for small regions, can connect to 10 edge devices, allowing data transmission every 10 min. This setup limits the capability for faster or real-time data analysis, which is a consideration for applications requiring more immediate data processing.

As can be seen, in contrast to the previously cited articles, this research introduces a novel approach to data collection and integration within public lighting infrastructure. While many existing studies focused on specific sensor types or communication methods, LoRaCELL employs edge devices to collect a wide range of data, encompassing current, voltage, lighting, UV index, sound, and more. Furthermore, our system utilizes LoRa N2P (N to Peer) communication for efficient data transmission to Amazon Web Services (AWS), where data are seamlessly integrated with MongoDB for storage. Additionally, LoRaCELL leverages Apache Spark for robust data processing and analysis, enhancing the insights derived from the collected information. Finally, using Power BI Pro v2.214 for data visualization and interpretation provides stakeholders with a comprehensive and user-friendly interface.

## 3. Materials and Methods

### 3.1. Proposed Method LoRaCELL

**Figure 1:**
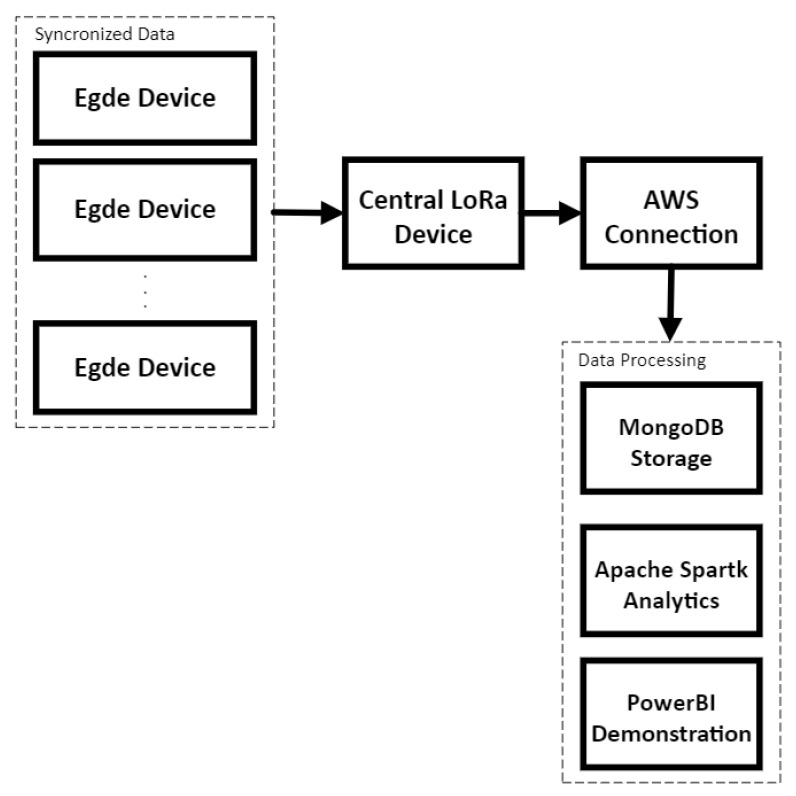
LoRaCELL schematics for data collection and processing workflow.

Figure 1 provides a schematic illustration of the methodology adopted in this study. The methodology revolves around a network of sensor devices (i.e., edges) to gather the data. The edges are interconnected with a central LoRa device that serves as a data aggregation and transmission hub. In order to ensure the robustness of data collection and minimize packet loss, the edges are synchronized with the central LoRa device. Note that this synchronization mechanism allows each edge to transmit data at fixed intervals, preventing collisions and ensuring seamless communication. Once the central device aggregates the data from various edges, they are transmitted to the AWS (Amazon Web Services) platform over the Internet.

Upon reaching AWS, the data are stored in MongoDB, a versatile and scalable database solution. This centralized storage ensures data integrity and facilitates efficient retrieval and analysis. The Spark Apache framework is employed for in-depth data processing and analytics. Spark Apache’s distributed computing capabilities enable handling large datasets, making it an ideal choice for this methodology. To transform the raw data into actionable insights, Power BI is utilized for data visualization. Through interactive dashboards and detailed reports, stakeholders can clearly understand the data patterns, trends, and anomalies. This visualization aids in informed decision-making, ensuring urban planning and management can be based on concrete data-driven insights.

### 3.2. Experimental Setup and Configuration

The approach of mini LoRaWAN transmission cells consists of standard edge devices with subtle modifications in their LoRaWAN protocol firmware, enabling communication with a simpler gateway, typically formed with just one communication channel to integrate the devices. The LoRaCELL concept is a star network with N2P, where multiple measuring devices transmit to a simplified one-channel gateway, combining their measurements and directing them to a standard LoRaWAN server on the Internet.

**Figure 2:**
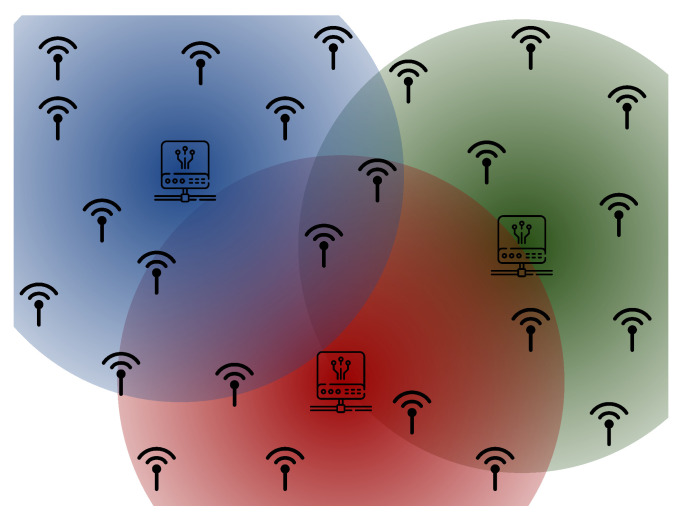
Basic structure of the LoRaCELL arrangement.

A functional structure of this configuration can be seen in Figure 2. This way, each edge device communicates with a central device, where devices in the Blue Ray communicate with the gateway in their region. Devices that share regions, as illustrated in the intersections of gateway boundaries, will only communicate with one device, as the bands are different for each central device. This approach ensures the well-known security of the LoRaWAN protocol with ABP, OTAA, and other standardized functionalities in systems without compromising performance but with a significant cost reduction for use in small areas (like super blocks or condominiums) with simple installation.

**Figure 3:**
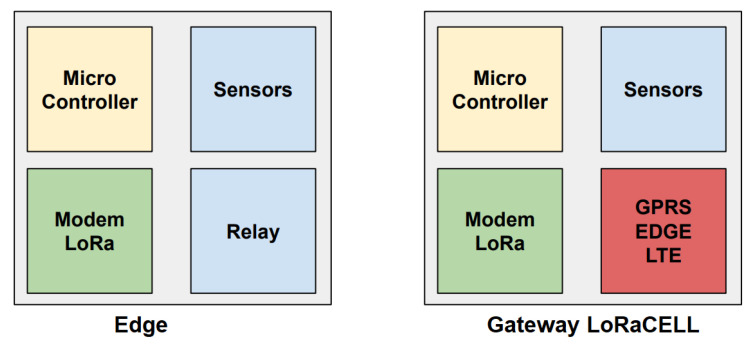
Basic difference between the edge device and the gateway with LoRaCELL.

Note that this approach differs from the original LoRaWAN once it implements a single LoRa channel per gateway, maintains a limited number of edge devices per gateway (typically fewer than 30), and covers only 10% of the area compared to the standard LoRaWAN approach. Moreover, the commercial value of the gateway sees a reduction, lowered by a factor of 100. The LoRaCELL gateway does not exceed USD30.00 in its assembly, compared to the LoRaWAN gateway at USD3000.00 for 5000 edge devices in Brazil. Thus, the LoRaCELL system has only one hardware for its devices; the gateway is an edge device with the addition of a GPRS, edge, or LTE radio for sending the data collected from other devices via the cellular network. Figure 3 illustrates this difference.

Streetlights will have edge devices installed throughout the monitoring region, connected to LoRaCELL gateways, combining the readings and sending them to the Internet via the cellular network. The main advantages of LoRaCELL can be summarized in the following topics:

- Unified hardware for all devices, whether edge or gateway;
- Compatibility for data transmission to existing LoRaWAN servers in the market;
- Low hardware cost;
- Suitable for use in small areas, such as residential blocks or condominiums;
- Supports multiple gateways per region, aiming at the cellular communication concept;
- Low packet loss compared to mesh approaches.

#### 3.2.1. Synchronization

Various techniques can be employed to synchronize IoT devices that utilize LoRa. These include using adjusted transmission windows, multiple access techniques, and more. These techniques aim to ensure that data packets do not collide and are all successfully received.

Time Division Multiple Access (TDMA) [27,28] is a channel access technique that divides time into fixed intervals and assigns each interval to a specific device. This method ensures that only one device transmits at a time, thus eliminating the possibility of collisions. However, TDMA requires devices to have precise and synchronized clocks to prevent overlapping their respective transmission windows, which can lead to interference and data loss. Achieving and maintaining this level of synchronization can be particularly challenging in environments with variable signal delays, such as in mobile or satellite communications. Additionally, the complexity of preserving synchronization increases with the number of devices in the network, potentially leading to scalability issues.

An alternative to TDMA is the use of random access algorithms, such as ALOHA [29] and CSMA (Carrier Sense Multiple Access) [30]. These algorithms allow devices to transmit whenever they have data to send, but with a strategy to handle collisions. For instance, in ALOHA, after a collision, the devices involved in the collision will wait for a random period before attempting to retransmit, thus reducing the likelihood of another collision. In CSMA, devices first “listen” to the channel to check if it is free before initiating a transmission, decreasing the likelihood of collisions.

When applying ALOHA and CSMA, LoRa Heltec begins to interrupt communication while another device is transmitting, causing the loss of the original and new packets. Furthermore, the devices sometimes restart after collisions, losing all stored sensor data. Therefore, a strategy that avoids collisions and meets the requirement of sending sensor data at each fixed time interval is needed.

The choice of a hybrid methodology allows taking advantage of the TDMA qualities of simple communication with defined spaces of time and solving the issue of the absence of a time clock, which is characteristic of other methods. Thus, there is a random synchronization phase and a part with fixed intervals for sending the message.

Within this project’s scope, the message-sending devices do not have access to a precise clock, equivalent to what they would have with a GPS sensor. Moreover, the possibility of power failures or shutdowns can result in a loss of system synchronization. Additionally, the random access approach, tested at the project’s outset, resulted in inconsistent and unsatisfactory performance for the project’s objectives. Therefore, a hybrid strategy was developed to synchronize the clocks of all devices. This approach was modeled based on the observation that, to avoid message collisions, each message should have a minimum interval of 28 s between them. Therefore, each edge device must send messages in a time window exceeding this interval.

**Figure 4:**
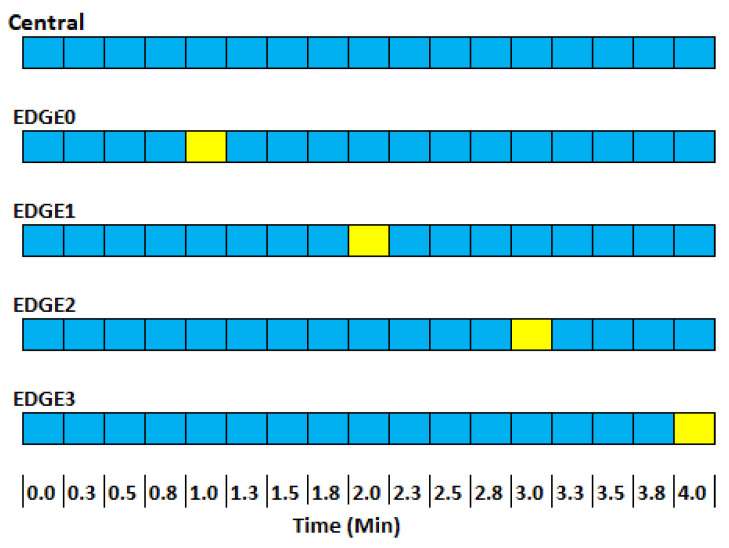
Communication pattern between edge and central devices.

At this project stage, each central device is connected to five edge devices programmed to send their readings every five minutes. The time intervals for message transmission were one minute to avoid collisions. A value substantially higher than the 28 s limit defined in the tests. Furthermore, considering LoRa devices’ inability to send and receive messages csimultaneously, we developed a communication pattern where the central device constantly remains in reception mode (Figure 4—blue). In contrast, the edge devices switch to send mode every minute (yellow), transmitting the data collected by the sensors. Although all devices in reception mode can see this message, only the central device will process the information, as each message contains metadata indicating which device the message should be directed to.

**Figure 5:**
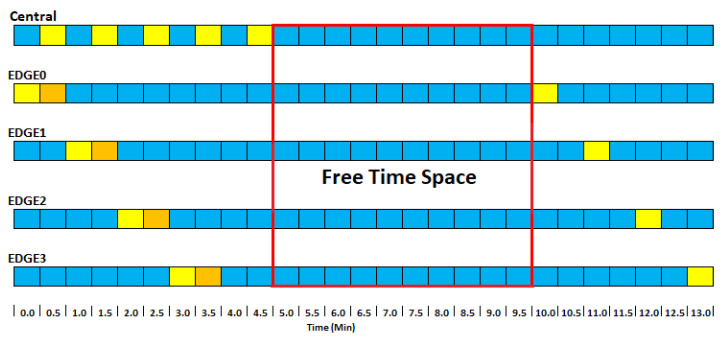
Synchronization between edge and central devices.

However, this synchronization process assumes that all devices are aware of real time, which is not true for the edge devices. Since only the central has Internet access and the updated time, we developed a strategy to allow the edge devices to synchronize (Figure 5).

Figure 5 depicts the synchronization pattern between the devices. The central accesses real time due to its Internet connection. The five edges will send messages at random times. One of them, the central, will receive and call edge0, returning the edge code to it and the delay time to send its first message. If it receives code 0, it will wait until its time reaches 10 min. The next message will be at 40 min. After that, the four edges will send messages at random times. The central will receive and call edge1, returning the edge code to it in real time. If it receives code 1, it will wait until its time reaches 11 min. The next will be at 21 min. This will continue until all devices are synchronized. Note that the central switches to send mode shortly after receiving the edge synchronization message. It then returns to receive mode to receive sensor data and other synchronizations. The free time space is the time between synchronizing and sending the first messages. This space was designed so that, even with the greatest conflict between synchronization messages, all devices are synchronized after a time. The yellow blocks show times when devices are sending messages over LoRa, while the orange blocks show when they are ready to receive messages only. The blue ones are when they are available for both cases.

During the synchronization process, the edges will not be sending sensor messages, only the necessary data for synchronization, and they will receive their edge code (from 0 to 4) in real time. Upon receiving this in real time, the device can now send synchronized messages.

If the central does not receive messages from one of the edge devices over 20 min (i.e., four transmission cycles), it is assumed that the device has stopped working or has even been replaced. In this case, the device’s record is deleted from the central, and the device will have to undergo the synchronization process again.

#### 3.2.2. Sensoring

For the sensing components, various sensors were employed to capture a wide range of environmental and electrical parameters. These sensors were chosen based on their accuracy, reliability, and compatibility with the LoRa Heltec V3 devices. Together, they provide a comprehensive data collection system for the project’s objectives:

- TSL2561: An illumination sensor designed to measure the intensity of visible light.
- MQ-135: A sensor for air quality measurements that can detect various gases.
- DHT22: A sensor for measuring humidity and temperature.
- Bmp280: A barometer and temperature sensor useful for atmospheric pressure measurements.
- MAX4466: A sound sensor was employed in this project to detect rain and traffic noise.
- ZMPT101B: A voltmeter for electrical measurements.
- UV ML8511: A sensor designed to measure solar ultraviolet radiation.
- ACS712 5A: An ammeter measuring up to 5 amperes.

The sensors deployed in this project were configured to take readings at regular intervals every 10 s. Given the nature of the data collection and the need to efficiently transmit these data, especially considering that there are ten edge devices connected to each gateway, sending every individual reading is not feasible. Instead, the data are transmitted every 10 min. A moving average of the sensor readings is calculated to represent the data collected during these 10 min intervals visually.

The moving average is a statistical calculation used to analyze data points by creating a series of averages of different subsets of the complete dataset. It is particularly useful as it helps smooth out short-term fluctuations and highlight longer-term trends or cycles. Equation (1) gives the moving average.

(1) $$MA\left(t\right)=\frac{1}{n}\sum_{i=0}^{n-1}x\left(t-i\right)$$

where MA(t) is the moving average at time *t*, *n* is the number of data points considered in the moving average (i.e., the number of readings taken during the 10 min interval), and x(t−i) is the sensor reading at time t−i.

In the context of our project, since readings are taken every 10 s for 10 min, *n* would be 60 (i.e., six readings per minute for 10 min). The moving average will then represent the average of these 60 readings, providing a single data point that captures the essence of the readings over the 10 min interval. This approach ensures that the data transmitted to the gateway are representative of the actual environmental conditions and efficient in terms of the amount of data being sent, thus optimizing bandwidth and storage.

#### 3.2.3. LoRa N2P

The primary advantage of LoRa is its ability to provide long-range communication with minimal power consumption. It achieves this through a unique spread spectrum modulation capable of demodulating signals below the noise level, allowing for transmissions over long distances.

The star network topology is one of the most common architectures used in LoRaWAN deployments. In this setup, multiple end nodes (often referred to as “devices” or “edges”) communicate directly with a central gateway. This direct communication eliminates the need for routing, making the network simpler and more scalable.

The star network’s N2P variation emphasizes the direct communication between any node (N) and its peers. In this context, a “peer” could be another end node or the central gateway. The N2P architecture is particularly beneficial for applications where devices need to exchange information among themselves, not just with the central gateway. This can be useful in scenarios where local decision-making or data aggregation is required before sending information to the central server.

**Figure 6:**
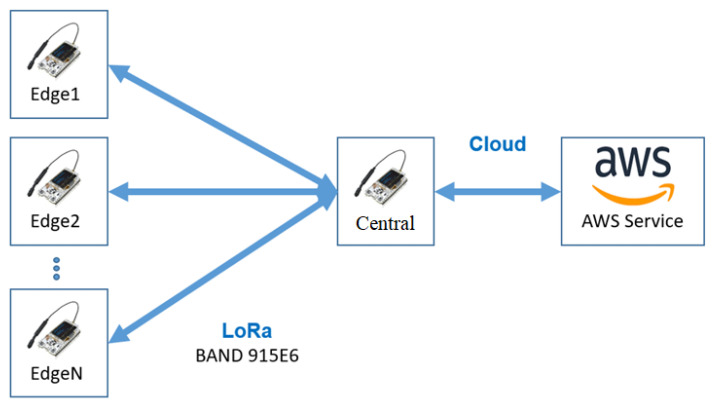
LoRa N2P communication.

Figure 6 shows the communication structure of LoRa N2P in this application. Note that it has N edge devices sending JSON (JavaScript Object Notation) to the central device. The central device receives these data and sends them to Amazon’s AWS service. The chosen connection method for linking the gateway and the AWS is GPRS. Although other communication methods like 4G LTE or 5G are possible, their implementation would increase the contract cost for the customer without offering significant improvements in our data transmission structure. Data, limited to JSON files of a maximum of 12 kB, are sent through the gateway every minute. Given the modest size of these files, the GPRS data transfer speed (80 Kbps) is sufficient to ensure timely delivery without incurring higher costs.

Configuring a LoRa transceiver involves adjusting parameters like transmission frequency, output power, bandwidth, spreading factor, and coding rate. These settings impact data transfer rates, ranging from 253 kbit/s to as low as 11 bits per second, affecting range and noise immunity. LoRa uses chirp spread spectrum modulation, enhancing noise resistance, especially in low-power transmissions. Bandwidth options can change, influencing data rates and transmission range. The spreading factor extends data over multiple symbols, improving noise immunity and range. The coding rate determines error correction levels, balancing data rate and reliability. Different coding rates allow LoRa devices to communicate effectively in various interference environments.

Configuring a LoRa transceiver requires the precise determination of several key parameters that significantly influence its operational behavior. These parameters include the transmission frequency, output power, bandwidth, spreading factor, and coding rate. LoRa’s modulation scheme offers flexibility, with potential data transfer rates reaching 253 kbit/s. However, by adjusting specific parameters, the data rate can be reduced to as low as 11 bits per second, enhancing the receiver’s processing gain and extending the transmission range.

LoRa’s chirp spread spectrum modulation intentionally broadens the signal across a more extensive bandwidth than is strictly required. This approach enhances frequency-domain noise immunity, especially for low-output power transmissions. LoRa supports bandwidths ranging from 7.8 KHz to 500 KHz in sub-gigahertz bands and 250 KHz to 1.6 MHz in the 2.4 GHz band. Typically, a narrower bandwidth yields a slower transfer rate but an extended range, while a broader bandwidth offers a higher data rate at the expense of range.

The chirp spread spectrum of LoRa disperses each payload data bit across multiple symbols, enhancing time-domain noise immunity and providing a processing gain. The spreading factor determines the temporal dispersion of each data bit. LoRa supports spreading factors between five and twelve, with only factors six through twelve accessible in the sub-gigahertz band. Notably, a factor of six often necessitates a TXCO for stability.

Distinct from the bandwidth and spreading factors, which influence the modulation scheme’s physical parameters, the LoRa coding rate governs the forward error correction added to the payload data. A higher coding rate enhances link reliability in interference but reduces the data rate. LoRa supports four distinct coding rates, each representing the ratio of actual data to error-correcting data. When selecting a coding rate, weighing the trade-off between the data rate and reliability is crucial. Notably, LoRa transceivers with different coding rates can still decode each other’s signals, allowing asymmetric configurations to optimize transfer rates in varying interference environments. Table 2 values were used for this application.

By configuring these parameters, a data rate of 7.81 kbps could be achieved [31]. Despite the relatively low transmission speed, this configuration offers enhanced reliability regarding device-to-device distance. Consequently, connecting more street lamps to the same switchboard becomes feasible, even though it necessitates longer time intervals between messages compared to the one-minute intervals in other scenarios.

The adaptability in configuring LoRa parameters such as bandwidth, spreading factor, and coding rate is key to optimizing system performance for specific project needs. This flexibility is particularly beneficial in urban environments with varying transmission conditions.

For instance, adjusting the coding rate to enhance link reliability can be more advantageous than maximizing the data rate in high-density areas or where interference is a concern. This approach is crucial for connecting numerous street lamps to a single centralizer, ensuring successful message delivery over longer distances or in challenging conditions.

Moreover, the ability to fine-tune bandwidth and spreading factors allows the system to adapt to different urban scenarios. A higher spreading factor can extend the range in areas with larger device distances. In contrast, a lower range setting might suffice in compact areas, enabling higher data transmission rates.

This flexibility improves the overall system efficiency and allows network customization to meet specific demands across different urban project areas. It results in a more robust and efficient system that adapts to environmental changes and specific local requirements. It enhances the project’s success in efficiently connecting and monitoring a wide network of devices, like street lamps, in a dynamic urban setting.

The structure of the message sent is a JSON with the format shown in Equation (2). The data transmitted by the edge device comprises several fields, each serving a specific purpose. The initial fields are dedicated to device identification, while the subsequent fields pertain to sensor readings.

- Name_Edge: This is a user-defined identifier the operator assigns to distinguish the edge device.
- E_ID: The Media Access Control (MAC) address is a unique hardware identifier for the edge device.
- F_ID: This integer value ensures that the edge consistently communicates with the intended central device, obviating the need to recognize the MAC of the central.

Combining the edge name and MAC address ensures the uniqueness and accurate identification of each device within the network. The subsequent fields encapsulate the readings from various sensors integrated into the edge device:

- T1 and T2: Represent the ambient temperature and the temperature on the luminary blade, respectively.
- U: Denotes the ambient humidity.
- G: Provides readings from gas sensors, specifically measuring carbon monoxide levels.
- Sound: Captures audio data using a microphone sensor. The data are processed using the fast Fourier transform (FFT) to extract values at specific frequencies.
- Light Sensor: Measures luminance in lux emitted from the lamp.
- ATM: Indicates the atmospheric pressure.
- UV: Represents the ultraviolet index.
- V and I: Denote the device’s voltage and current readings, respectively, measured in volts and milliamps.
- lat and lon: Specify the latitude and longitude positions of the device, respectively. Considering it is a stationary entity, these values are set during the edge device registration.

This structured data format ensures a comprehensive and organized representation of device identification and sensor readings, facilitating efficient data processing and analysis.

(2) $$Json=\{\begin{array}{c} Name\_Edge=Edge\_0,\\ E\_ID:.DC:54:75:C7:FD:F0,\\ F\_ID.:0,\\ T1:25.2,\\ U:31.4,\\ G:192,\\ Sound:12.2,\\ Light:530,\\ T2:28.4,\\ ATM:9200,\\ V:221.1,\\ I:450.1,\\ UV:3.4,\\ lat:-21.77784,\\ lon:-43.37254 \end{array}\}$$

### 3.3. AWS—MongoDB—Power BI

A Python script was developed in the initial project phase to establish connections with essential services and execute the requisite operations. This script operates seamlessly within the Amazon Elastic Compute Cloud (Amazon EC2 [32]) environment, functioning as a virtual machine. This strategic choice eliminates the need for dedicated physical computing resources.

During the project, a critical decision was made regarding selecting a database. After extensive research, it was concluded that a NoSQL database architecture would be the most appropriate choice due to the dynamic nature of the data and the need for flexibility in handling various data types [33].

The decision was further supported by the strong integration capabilities of MongoDB [34], which works seamlessly with Apache Spark [35] and Power BI [36] to streamline data processing and visualization. This integration was essential in ensuring efficient data management and providing real-time insights.

Additionally, the preference for Amazon EC2 as the hosting environment was rooted in the reliance on AWS as a core component of the smart city solution. With AWS IoT Core as the primary IoT platform, Amazon EC2 seamlessly integrated into the cloud-based architecture, ensuring compatibility and simplifying the overall system.

In deploying the LoRaCELL application, the choice for Amazon Web Services (AWS) was primarily influenced by two critical factors: its seamless integration with existing software infrastructures, particularly MongoDB, and its cost-effective pricing model. The compatibility of AWS with MongoDB, a core component of our data management system, ensures uninterrupted and efficient database operations, a crucial aspect of the application’s performance. Furthermore, the financial aspect of this decision cannot be understated. AWS presents a budget-friendly solution, especially significant for our project’s scope. Its scalable pricing strategy, which allows payment proportionate to the resources used, aligns perfectly with the varying demands of the LoRaCELL application. While other cloud services like Microsoft Azure and Google Cloud Platform offer similar capabilities, the combination of MongoDB integration and a more advantageous cost structure under AWS makes it the most suitable choice for our specific requirements. However, this preference for AWS includes the potential future consideration of alternative providers should our operational needs or budgetary conditions evolve.

The MongoDB database effortlessly connects to Power BI software to facilitate data visualization. This connection is established through MongoDB’s “Connector for BI” service, enabling us to harness the data visualization and analysis potential for the smart city solution fully.

After acquiring sensor data, it will initiate a refinement utilizing the Apache Spark framework [37]. This process will involve transforming the raw data into useful information, which will be used to monitor streetlight conditions. Moreover, the project aims to use these frameworks to enhance public safety, urban mobility, and other aspects that can improve the overall quality of life of the population.

The first idea evaluated was to analyze actual electricity consumption by calculating power usage. Furthermore, the hour meter will track when the streetlights are on and off. In case of “dayburn”, the system will detect the absence of current and promptly report the information.

To ensure compliance with the voltage standards set by ANEEL (the National Agency of Electric Energy) [38], it will use the acceptable voltage variation as a benchmark to notify of any deviations in streetlight voltage from the required specifications. In line with this approach, it will also integrate a lifespan management system into the project.

Considering the community’s well-being, sound and gas sensors will collaborate to provide valuable information to claim insights into air quality. All applications mentioned are available in more detail in Table 3.

**Figure 7:**
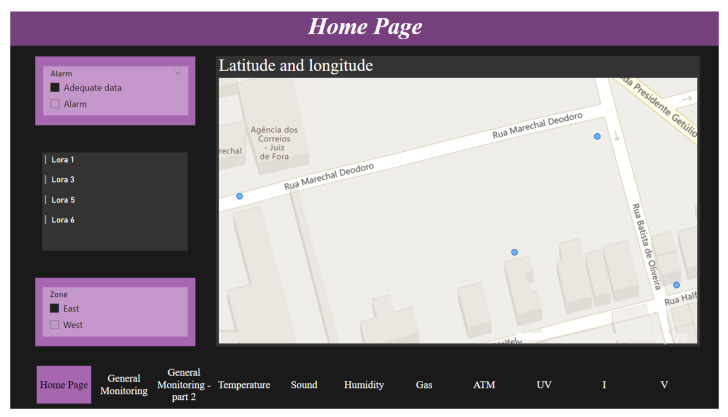
Dashboard on Power BI. Note that the blue points indicate where the LoRaCELL-driven smart lighting system is installed.

Not all data will necessarily pass through Apache Spark. Nonetheless, these data and the treated ones will appear in a dashboard (Figure 7) created with the assistance of Power BI [39]. The first page presents a menu page and indicates the location of streetlights, showing the points with a filter of the zone and the occurrence of problems. If there is an alarm, the dot in the map will change color from blue to red.

Further, there is also a general monitoring page that shows the identification information of the edge and its respective real-time measures of sensors. The remaining pages were implemented with a graph presenting each of these data points.

Crucially, insights derived from Apache Spark are integrated into the dashboard, ensuring a holistic view of street lighting conditions and environmental data for effective decision-making.

### 3.4. Materials

Different materials were employed to facilitate effective data collection and communication to enhance the project’s development and implementation. The LoRa Heltec V3 devices were selected for the edges and gateways due to their proven reliability and outstanding performance in IoT projects. Table 4 presents the individual product costs as of September 2023. Note that these prices apply to single-item purchases and do not include bulk purchase discounts. The quotations took place in Juiz de Fora, Brazil, mainly in online stores such as CurtoCircuito.com.br.

**Figure 8:**
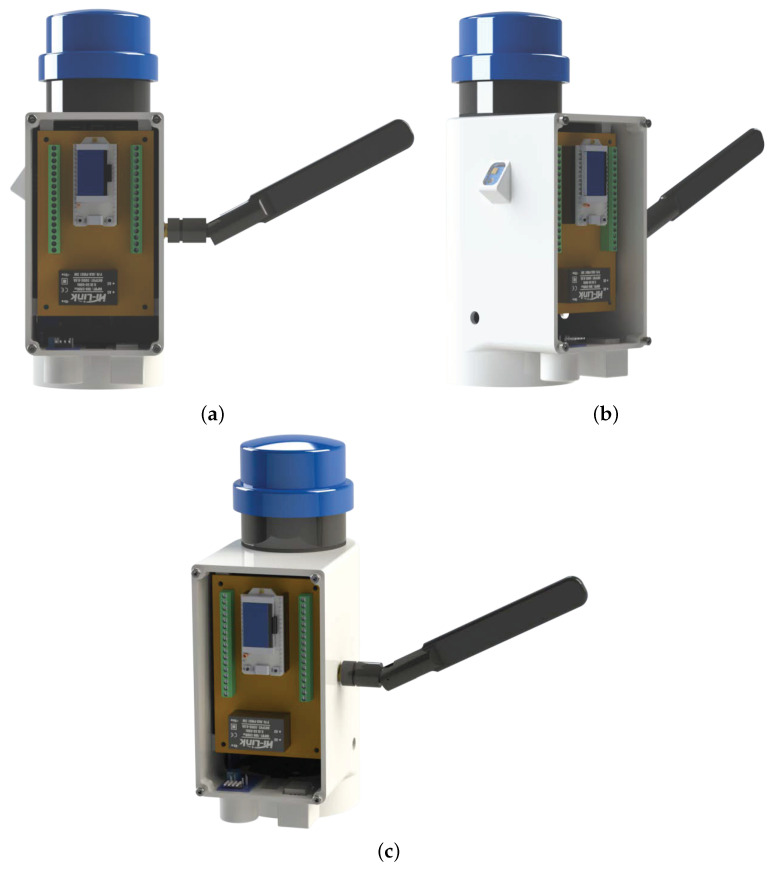
Front, right-side, and left-side views of the product. (**a**) Front view of components. (**b**) Left view of components. (**c**) Right view of components.

**Figure 9:**
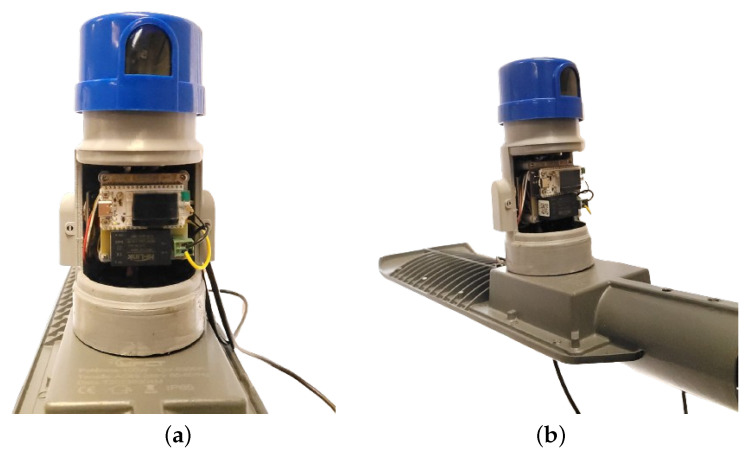
Front and side views of the real prototype. (**a**) Front view of prototype. (**b**) Side view of prototype.

Our components were adjusted according to Figure 8a–c, which display the front, left-side, and right-side views of the product. The assembly video can be viewed at https://youtu.be/sY8itxGi960 (accessed on 27 September 2023). The prototype used in the tests is shown in Figure 9a,b with its front and side views, highlighting the presence of the photoelectric relay, which is already available in the lamp. The lamp used for testing has a power of 100 W, and we use the suppliers “Brisa” and “YPCT”. The results in the Section 4.1 are with the supplier YPCT.

We have a three-way connector for a photoelectric relay on the bottom of the equipment, which is compatible with most luminaires without dimming. In addition, the DHT22 (temperature and humidity) and MQ-135 (air quality) sensors are connected in the two cavities below to allow access to environmental information without being affected by rain or other factors that could impair their operation. On the left side of the device, we have the ML8511 (ultraviolet) sensor that measures the UV index, enabling the calculation of the time of day based on its operation.

The ZMPT101B (voltmeter) and ACS712 5A (ammeter) sensors are connected inside the device, as they are connected to the cables between the upper and lower three-pin connectors. This way, we have the necessary insulation to avoid problems in measuring voltage and current.

The TSL2561 (illumination) sensor is coupled to the bottom of the luminaire, allowing it to capture its operation and the amount of illumination it delivers. The Bmp280 (temperature and pressure) sensor is attached to the upper fin of the luminaire, enabling the calculation of its temperature and predicting malfunction if the temperature exceeds its operating range.

Above the equipment, a photoelectric relay isolates the lamp’s operation from the measurement equipment. In newer models, the lamp’s activation will be added to LoRaCELL.

## 4. Results and Discussion

### 4.1. Results

In order to test the item synchronization proposal, a time experiment was carried out for each device to synchronize. The test is based on the time between sending the message (i.e., the time that the edge device sends the encrypted message) until synchronization processing (i.e., the central device receives the message, performs its decryption, performs the processing, and sends the message encrypted sync). This way, after receiving the encrypted message, the edge device removes the encryption and synchronizes, at which point the synchronization time is calculated.

However, this approach raises questions about the efficiency and reliability of the synchronization process, especially in scenarios with high network traffic or interference.

**Figure 10:**
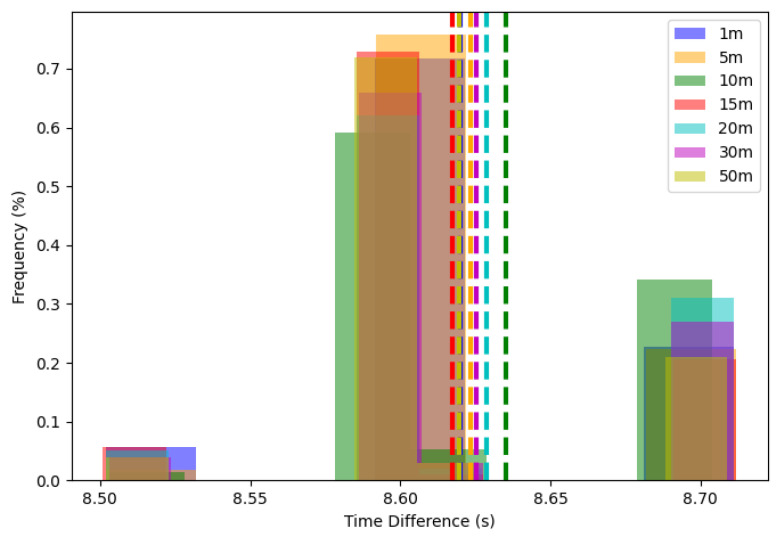
Communication between times of sync message and distance of central and edge devices.

For the test, different distances between the devices were tested, allowing better visualization of the data and the impact of distance on synchronization time. Figure 10 shows these results.

Figure 10 shows that regardless of the distance between the devices, most of the messages had values close to 8.6 s. Thus, the transmission rate, related to the values in Table 2, remained constant. This consistency, while impressive, might not accurately represent real-world conditions, where environmental factors could significantly impact transmission times. For the test, devices were added at measured distances without walls or any structure that could delay the messages. A total of 200 messages were sent at each distance, achieving 100% of communication. The averages, corresponding to the dashed lines, are very close, ranging between 8.6 s and 8.65 s.

**Figure 11:**
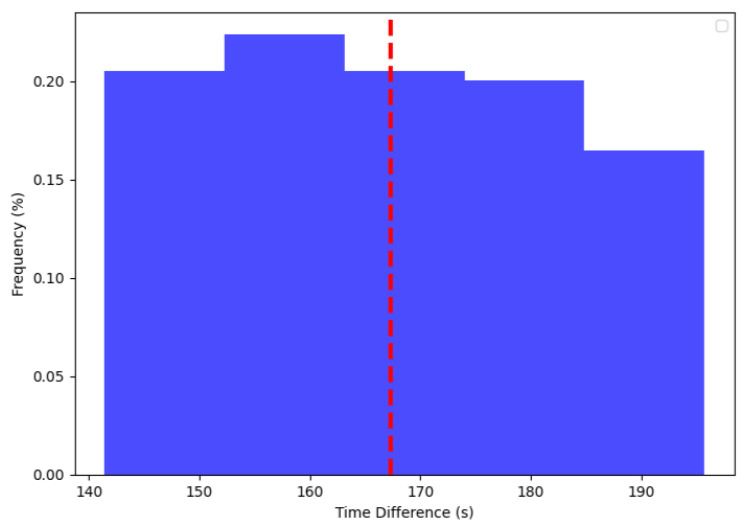
Time between all sync edges.

Figure 11 gives the synchronization time for devices at various distances. In this case, it represents the time for synchronizing the ten edge devices. Two devices were placed at 1 m, two at 5 m, two at 10 m, one at 15 m, one at 20 m, one at 30 m, and one at 50 m. Note that there was a greater variation in the total synchronization time of the devices, ranging from 140 s to 200 s. The explanation for this variation is that in some simulations, the faster ones, all messages were sent and arrived at their devices on time without two or more edges sending messages simultaneously. In contrast, the slower simulations experienced some conflicts between messages, causing the central device not to receive the message from any device. Therefore, as the next message was randomly between 30 and 60 s, this delay occurred for the subsequent message. Despite the greater variation, all devices were synchronized within 200 s, enabling them to send sensor messages at the correct times.

**Figure 12:**
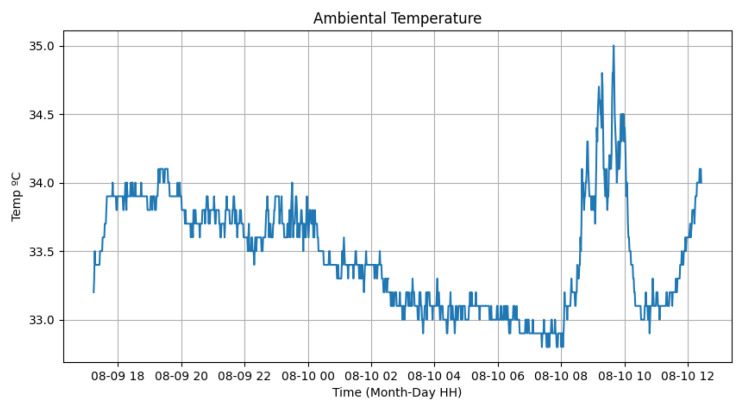
Ambiental temperature sensor test.

**Figure 13:**
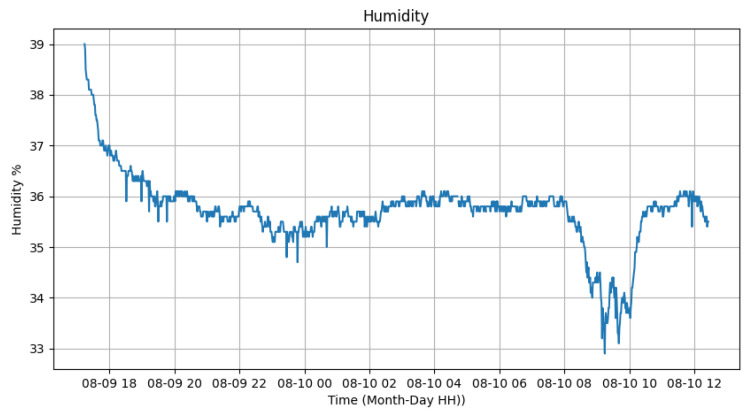
Humidity sensor test.

In relation to the sensors, we conducted tests from 6 p.m. on 18 September 2023 until 12 p.m. on 19 September 2023. The tests began with the automatic activation of the luminaires by the photovoltaic relay at 6 p.m. and continued until their deactivation at 6:30 a.m. the following day. These tests were conducted in the GRIN (Intelligent Robotics Group) laboratory at the Federal University of Juiz de Fora. Figure 12 and Figure 13 display the variation in ambient temperature and humidity measured by the DHT22 sensor. It is noticeable that the ambient temperature had little variation (between 33 ºC and 35 ºC) during the test, since it was a controlled environment without sunlight. Meanwhile, the humidity varied from 33% to 38%, demonstrating that the sensor could measure these data. Data transmission was performed every 1 min.

**Figure 14:**
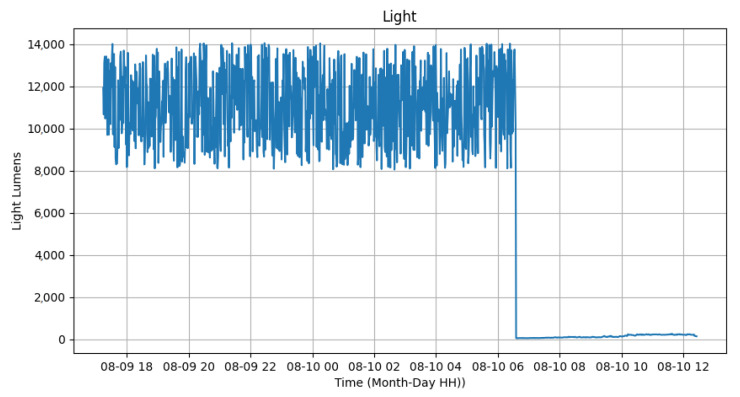
Light sensor test.

Figure 14 presents the results of the light sensor located at the bottom of the luminary. While the light was on, the equipment detected luminosity ranging from 8000 to 14,000 lumens. Despite the lack of consistency in these values, varying significantly within this range, when the photovoltaic relay turned off the luminary, the value dropped to between 50 and 70 lumens around 6:30 a.m. By noon, the value increased due to the reflectance of materials with sunlight but did not exceed 350 lumens. Therefore, it is possible to use the illumination sensor (TSL2561) to identify the operation of the lamp, as it is significantly higher than the external interference from other lamps and the sun.

**Figure 15:**
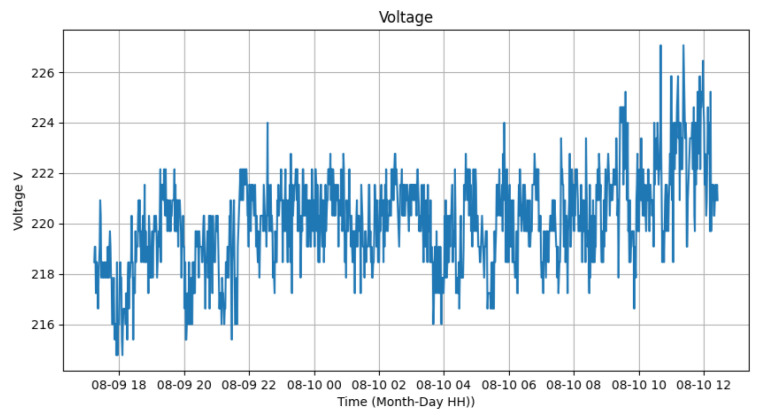
Voltage sensor test.

**Figure 16:**
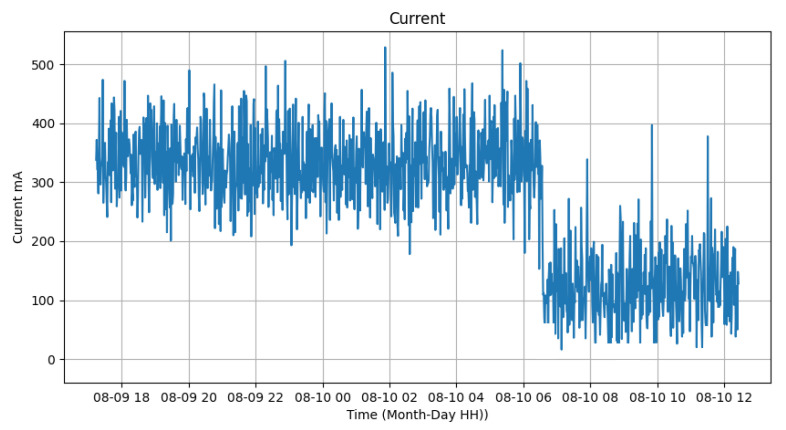
Current sensor test.

**Figure 17:**
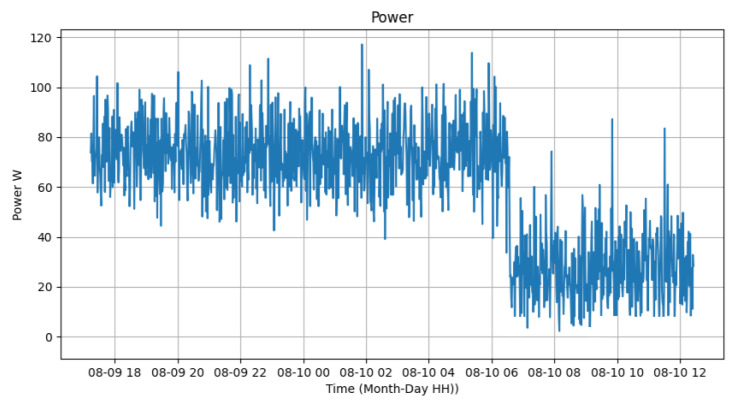
Power test.

Figure 15, Figure 16 and Figure 17 represent the measured voltage and current, while the last one represents the electric power calculated from these two data. It can be observed that, despite the significant variation in voltage values, they are within the range of 216 V and 226 V. In the case of electric current, it is possible to analyze that the current varies between 200 and 500 mA while the lamp is on and between 30 and 250 mA while the lamp is off. Therefore, the instantaneous power of the device varies from 50 to 100 W while the luminary is on and from 5 to 40 W when the equipment is off. The total average consumption of the experiment was 1142.19 Wm, resulting in an average energy of 63 Wh. While the lamp was on, the consumption was 1004.35 W, and when off, it was 137.84 W. The averages varied from 71.74 Wh and 34.46 Wh for the luminary on and off, respectively. The sensors used were ZMPT101B for voltage and ACS712 for current.

**Figure 18:**
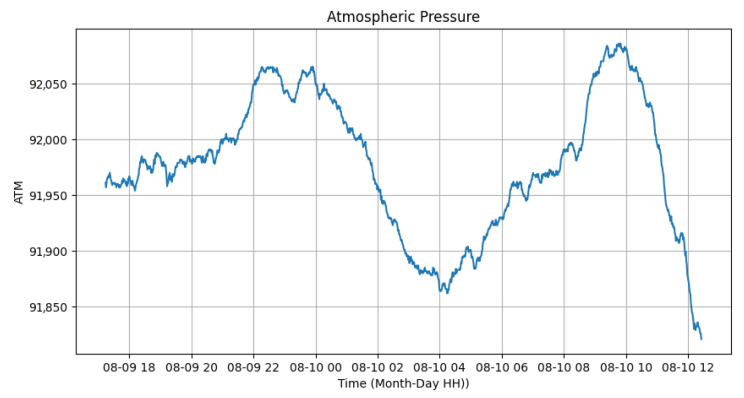
Atmospheric pressure test.

**Figure 19:**
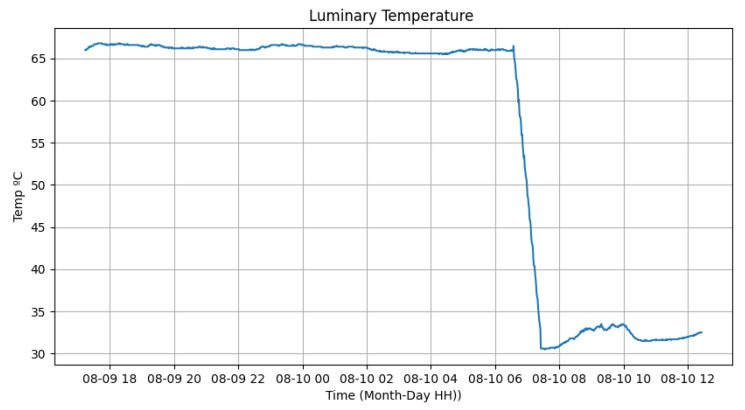
Luminary temperature test.

The atmospheric pressure and the temperature of the luminary, provided by the Bmp280 sensor located on the luminary fin, can be seen in Figure 18 and Figure 19. It is possible to identify that the temperature of the luminary when turned on had high values, close to 67 ºC. In contrast, when turned off, it varied from 30 to 34 ºC, allowing us to observe that the operation of the equipment generates significant heat from the device. Since the tests were conducted inside the laboratory, without air currents, the temperature of the luminary experienced slower drops, taking almost 1 h to decrease its temperature to near ambient. The atmospheric pressure varied from 91,850 Pascal to 92,050 Pascal, showing no variation in whether the luminary was on or off.

**Figure 20:**
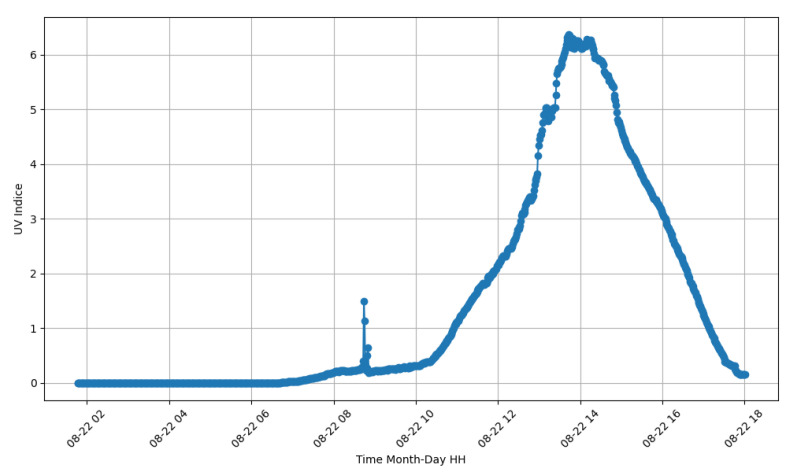
UV index sensor test.

Since the test of all sensors was conducted inside the laboratory, it was found that the variation in the ultraviolet sensor (ML8511) had no change. Therefore, another test of this sensor was carried out on 22 August 2023 from 2 a.m. to 6 p.m. The test can be seen in Figure 20. It is noticeable that with the variation in the day, starting at 6:30 a.m., the value began to increase, reaching its peak around 1 p.m. From 2 p.m., the UV index value decreased again, becoming zero at 6:30 p.m. These values can activate the lamp without needing a photoelectric relay for the same purpose.

With the results demonstrated earlier, it is possible to identify potential failures in the lamp. If the UV index is above a certain threshold (0.5) and the equipment’s current is above 200 milliamperes, the lamp is on during the day. The same can occur at night when the UV index is zero, the lamp’s current is zero, and the illumination is low, representing that the equipment has experienced some failure. Of course, there can be failures in the readings of the sensors. Still, suppose the history shows that any of these data are contradicting. It is possible, along with the latitude and longitude location of the lamp, to calculate an optimized maintenance route, allowing the maintenance teams to have a greater capacity to perform their task.

In addition to maintenance issues, it is possible to indicate other factors, such as the power consumed by the lamps. If an entire region has this system in all its luminaires, it is possible to calculate the energy consumed by public lighting, allowing for the renegotiation of contracts and even improving the company’s demand forecasting. Compared to other studies in the literature, LoRaCELL stands out due to its distinctive features. While LoRaCELL shows promise, it is important to critically evaluate its scalability and adaptability in diverse urban environments, which can present unique challenges.

### 4.2. Discussion

The previous results demonstrated our model’s ability to detect environmental and energy variables, starting with low implementation costs, as highlighted in Table 4, which allows scalability.

Compared to current technologies, the first point to highlight is the cost. LoRaWAN technology can connect several devices per gateway. However, the initial price of this device is high compared to the LoRaCELL gateway. In regions where many devices will be installed with a view to the gateway, LoRaWAN may be justified. But, in cases of several different cores, installing LoRaWan will be costly and waste a lot of processing capacity. In this case, LoRaCELL, with a smaller number of connected devices but at a low cost, is justified.

Compared to GPRS, 4G LTE, and 5G networks on all devices, as some of the references mentioned, adding more hardware within the product and the need for individual chips increases the cost of the solution. In this case, using LoRa communicating with a centralizer reduces the cost of the solution. As the data rate is low, in this case, the choice of a JSON every minute allows the centralizer to make this choice. With a higher data rate or even a system that requires real-time interventions, the choice of other technologies would be justified, but at a higher cost.

One of the most expensive sensors designed for the project was the GPS sensor (Global Positioning System). Adding this sensor would resolve the issue of the device knowing the world time, facilitating data synchronization. However, its location, set only when installing the device, would be unused information and increase unnecessary costs. In this way, hybrid synchronization was proposed, combining the need for windowing to avoid data collisions in the LoRa network with the random issue of the synchronization process. LoRaWAN allows several devices to send messages simultaneously, and synchronization is unnecessary on devices connected to cellular networks. However, as previously explained, the main objective is a low-cost method, requiring strategies to solve the problems of multiple devices trying to synchronize, for example, in a power outage.

The chosen sensors aim to transform each public lamp into a small weather station. Data such as temperature, humidity, and atmospheric pressure can be viewed by area, and there is no need for all devices to have these sensors.

In this way, the prototype developed was designed modularly, allowing three distinct models to exist: the first model, with all sensors, can measure environmental data, luminaires (illumination and temperature), and electrical quantities (current, voltage, etc.). The second model, still under development, will only have electrical and luminaire sensors, while the last model will only have electrical quantities.

The objective of creating these two other models is to study the luminaire and electrical quantities, the latter being the main objective of the company financing the project. In this way, two to three complete devices would be added per region to have all the environmental data, while in a subsampling per luminaire seller, they would have the lighting data. And in more devices, they would have the simplest model to calculate the electrical consumption of the luminaires.

Therefore, LoRaCELL aims to apply a low-cost method to monitor environmental, lighting, and electrical quantities. The proposals, compared to those available, have their advantages mainly due to the lack of need for a cellular network on all devices and their low cost compared to LoRaWAN, 4G LTE, and 5G.

## 5. Conclusions and Future Work

### 5.1. Conclusions

The developed system successfully synchronized with each other and sent IoT sensing data at a low cost compared to traditional technologies such as LoRaWAN. The synchronization process of LoRaCELL allowed devices to send messages with a certain level of security, enabling the reception of data from all sensors within the same bandwidth. Additionally, the connection allows for over-the-air (OTA) firmware updates, as the central device can send a message to all devices, preventing them from sending messages and only receiving them, thus updating the firmware without the need for on-site teams. This functionality is one of the future proposals for the advancement of research.

Another criterion is that the chosen sensors successfully implemented the ideas envisioned at the beginning of the project, such as the ability to detect faults in the lamp and calculate the average expenditure of the equipment. It is intended to optimize this hardware version for a more compact and reliable system, allowing greater security, isolation from environmental conditions, and higher efficiency in energy and data aspects.

### 5.2. Future Work

Future research will focus on enhancing the efficiency of data transmission and processing mechanisms, exploring new sensor technologies for more comprehensive data collection, and further improving the integration of various data sources. Developing innovative algorithms and machine learning techniques could also be important in optimizing energy consumption and predictive maintenance of public lighting systems.

Another significant aspect of our future work involves installing 300 devices in collaboration with CEB (Companhia Elétrica de Brasília) utilizing LoRaCELL technology. This initiative aims to enable data evaluation and hardware and software implementation for supervision in an electric energy company. The selection of sensors for this project is still under consideration, allowing for comprehensive environmental and electrical analysis in a real-city scenario.

The limitations of our proposed model are particularly about the number of devices connected to the same gateway. As our system is designed to send a message every minute, the more devices connected to a single gateway, the greater the interval between messages from each device. For instance, with ten devices connected to the same gateway, sensor data would be received only every 10 min. This setup poses challenges for analyzing variables that require real-time data. One solution would be to carry out the analysis within the Heltec LoRa device. However, these have limitations in processing capacity, in addition to issues such as alarms and alerts, which would have to wait for the message to be sent. Additionally, future data processing faces constraints in data storage capacity. An increased number of edge devices necessitates more extensive storage solutions.

Resolving these limitations will be crucial to our future work. We plan to investigate methods to efficiently manage larger volumes of connected devices without compromising data transmission frequency and real-time relevance. This might include optimizing gateway capabilities or exploring decentralized data processing approaches.

The evolution of the prototype into a product will require rain and weather resistance tests, allowing it to be added to a real electrical network. The external sensors will be added with connections proof against these conditions, while the internal ones will be tested against water spray, wind, and dust. Another concern is the issue of equipment configuration, where the original idea is plug-and-play equipment, allowing minimal configuration for the operator.

## Figures and Tables

**Table 1:** Related studies.

Article	Year	Method	Proposal
Rossi et al. [12]	2016	IoT Control System	Energy-efficient public lighting with 4G communication
Joy et al. [16]	2016	Mobile Data	Privacy-preserving data collection
Gehlot et al. [2]	2021	Long-Range IoT	Smart lampposts with Wi-Fi connection and mesh
Zhu et al. [14]	2022	IoT-sensing	Hybrid sensing: IoT-sensing and crowdsensing in a complementary manner
Anand et al. [15]	2022	Federated Learning	Smart street light monitoring with machine learning
Mante [17]	2023	oneM2M standards	IoT data processing
Povalac et al. [19]	2023	LoRaWAN Traffic	Identified security and regulatory issues
Kane et al. [20]	2023	Wi-Fi HaLow vs. LoRa	Wi-Fi HaLow’s promising performance
Farhad and Pyun [22]	2023	LoRaWAN with ML	ML’s potential in resource management
Giuliano et al. [24]	2023	Batteryless LoRa Sensors	Adaptive transmission algorithms
Kumar et al. [25]	2023	LoRa-based E-Nose	Networked gas sensor system
Andreadis et al. [26]	2023	IoT in Remote Areas	Synchronization protocol with UAV
This Article	2023	LoRaCELL	Low-cost system to observe energy-efficient and IoT data

**Table 2:** Parameter values used in the application.

Parameter	Value
Bandwidth	915.0
Spreading Factor	12
Coding Rate	8

**Table 3:** Methods utilized for each application.

Application	Method
Actual electricity consumption	$\mathrm{Total}\;\mathrm{Energy}\left(E\right)=\int_{t_{1}}^{t_{2}}P\left(t\right)dt$
Hour meter	if light > 500 lux, then “streetlight is on”
“Dayburn”	if (streetlight is on) and (6 < hour < 18)
	and (current > 100 mA), then “dayburn”
Voltage variation	if voltage > 220 · 1.005 or voltage < 220 · 0.995,
	then “voltage didn’t vary”
Lifespan management system	when ((light < 350 lux) and
	(hour > 18 or hour < 6) and (current > 400 mA)),
	then “change lamp”
	when ((light < 250) and (hour > 18 or hour < 6) and
	(current > 400 mA)), then “lamp burned”
Air and sound pollution	when (sound ≥ 70 dB) and (gas ≥ 0.5), then “pollution”

**Table 4:** Cost of equipment used in the project.

Equipment	Cost (USD)
LoRa Heltec V3	33.10
TSL2561 (Illumination)	33.10
MQ-135 (Air Quality Sensor)	4.01
DHT22 (Temperature and Humidity)	5.01
Bmp280 (Temperature and Pressure)	1.79
ZMPT101B (Voltmeter)	3.81
UV ML8511 (Ultraviolet)	6.62
ACS712 (Ammeter) 5A	2.71

## Data Availability

Data are contained within the article.

## Abbreviations

The following abbreviations are used in this manuscript:

Amazon EC2	Amazon Elastic Compute Cloud
ANEEL	National Agency of Electric Energy
AWS	Amazon Web Services
CSMA	Carrier Sense Multiple Access
FL	Federated Learning
IoT	Internet of Things
JSON	JavaScript Object Notation
LED	Light Emitting Diode
LoRa	Long Range
MAC	Media Access Control
ML	Machine Learning
N-IGSS	Networked Intelligent Gas Sensor System
N2P	N to Peer
OTA	Over-the-air
UAV	Unmanned Aerial Vehicle
TDMA	Time Division Multiple Access
